# Explainable Text-Based Depression and Suicide Risk Prediction from Social Media Using Deep Learning and Graph Neural Networks

**DOI:** 10.3390/healthcare14111440

**Published:** 2026-05-22

**Authors:** Atiq Ur Rehman, Abid Iqbal, Ali Sayyed, Zaheer Aslam, Muhammad Ismail Mohmand, Ghassan Husnain

**Affiliations:** 1 Department of Computer Science, CECOS University of IT and Emerging Sciences, Peshawar 25100, Pakistan; ateeq@cecos.edu.pk (A.U.R.); zaheeraslam@cecos.edu.pk (Z.A.); ghassan.husnain@gmail.com (G.H.); 2 Department of Computer Engineering, College of Computer Sciences and Information Technology, King Faisal University, Al-Ahsa 31982, Saudi Arabia; aaiqbal@kfu.edu.sa; 3 Department of Computer Science, FAST National University of Computer & Emerging Sciences, Peshawar 25100, Pakistan; ali.sayyed@nu.edu.pk; 4 Department of Computer Engineering, Faculty of Engineering and Natural Sciences, Istanbul Atlas University, Istanbul 34408, Turkey

**Keywords:** mental health surveillance, suicidal ideation detection, depression classification, BERT, Secretary Bird Optimization, Graph Neural Networks, explainable AI, SHAP

## Abstract

**Objectives:** The rise in the frequency of mental health concerns (depression and suicide) expressed on social media calls for reliable, explainable, and efficient computational methods for mental health surveillance. In this paper, we propose an interpretable framework for text-based detection of post- and community-level mental health risk on social media. **Methods:** The framework combines (i) Secretary Bird Optimization (SBO) for feature selection of informative linguistic and psychological features, (ii) a BERT (Bidirectional Encoder Representations from Transformers)—CNN (Convolutional Neural Network) model for post-level reasoning, and (iii) a Graph Neural Network (GraphSAGE) for community-level reasoning. The graph is estimated based on semantic similarity between posts and author relations, instead of social interactions (e.g., mentions, replies) between authors. We use SHAP and LIME for model interpretability, uncertainty, and calibration analysis to evaluate the trustworthiness of predictions. **Results:** The model delivers 93.1% accuracy, 0.91 F1-score, and 0.944 ROC-AUC on the eRisk and CLPsych datasets using a strict user-disjoint validation strategy. SBO lowers the number of features by about 38%, leading to better generalization. The graph-based model enables improved learning of post and user representations by capturing relational dependencies. **Conclusions:** Our approach offers an explainable and robust means of detecting mental health risk from text. Graph-based representations of semantic and authorship interactions enable community-level analyses, while interpretability and uncertainty estimation facilitate possible human-in-the-loop decision-making. This research does not explicitly consider a human-in-the-loop experiment.

## 1. Introduction

The evolution of social media has radically transformed the way individuals communicate or express emotions and seek support during times of mental distress. Twitter and Reddit allow mass expression and result in a high-volume stream of text, which, when properly utilized, can reinforce the work of public health surveillance by allowing the population-wide monitoring of mental health indicators. Nevertheless, one of the main methodological issues is to transform these enormous, unstructured, and noisy streams of data into credible psychological constructs. Mental health conditions are generally accepted as one of the critical public health issues in the world; according to the World Health Organization (WHO), hundreds of millions of individual’s experience depression, and a significant number of deaths occur every year due to suicide [1]. Such challenges can also be seen in student and young adult groups as the symptoms of anxiety, depression, and suicidal ideation are often common in these groups, which highlights the necessity of scaling and making screening methods more accessible [2]. Timely intervention and detection at an early age is vital, and many people do not receive conventional mental healthcare services because of stigma and financial or geographical access, as well as a lack of awareness [3]. In this respect, social media-based monitoring can serve as an auxiliary tool that would assist in detecting possible risk indicators at an earlier stage and aid in human-in-the-loop triage but should not be regarded as a part of clinical diagnosis and treatment trajectories.

With the widespread use of digital media, the use of Artificial Intelligence (AI) as a tool to identify mental health problems from written material has expanded rapidly in recent years. The traditional machine learning models, such as Support Vector Machines (SVM) and Random Forests, were reported to achieve moderate success in mental health classification [4,5]. However, they are often limited in mental health forums because the features are hand-engineered and not expressive enough for the semantic complexity or context nuances in figurative language commonly used in recovery-focused narratives. The introduction of deep learning with transformer architectures, and in particular Bidirectional Encoder Representations from Transformers (BERT) [6], has brought NLP to new frontiers with the ability for bidirectional context modeling. It can produce state-of-the-art results on tasks such as sentiment analysis, emotion recognition, and mental health classification [7]. When fine-tuned to domain-specific corpora, it can also capture subtle linguistic cues of distress and suicidal ideation apparent in casual or metaphorical language [8]. However, BERT processes individual posts and may therefore miss larger behavior patterns and relationships across user communities. A complementary approach that has emerged to compensate for this limitation is graph-based modeling. Graph Neural Networks (GNNs) can model the relational dependencies between users and posts, reflect how content propagates, and discover regularities in community evolution [9,10].

Recent research has investigated graph-based models for mental health analysis with various types of GNNs, such as Graph Convolutional Networks (GCN) and its variants. However, unlike these studies, this paper does not contribute to the introduction of graph-based modeling, but the integration of contextual text representation, bio-inspired feature learning, and inductive graph reasoning in a single, reproducible framework. Moreover, this study utilizes GraphSAGE given its inductive nature, which enables the inference of unseen nodes. This feature is crucial for user-disjoint evaluation, where unseen test users do not exist during training, and allows for more realistic off-the-shelf deployment in contrast to transductive approaches such as GCN or GAT.

In contrast to the success of both transformer-based and graph-based methods, two significant challenges remain, which consist of (1) efficient feature processing on high-dimensional and noisy spaces created by social media data, and (2) powerful learning results that are well generalized as well as robust. These tasks are well-known to bio-inspired metaheuristic algorithms that have shown successful techniques for feature selection and hyperparameter tuning. A very important algorithm, which has demonstrated its high performance in combining exploration and exploitation that it is of particular importance for selecting the most useful discriminative features in a high-dimensional background, is the Secretary Bird Optimization (SBO) algorithm [11]. When integrated into deep learning pipelines, SBO can benefit those with improvements in accuracy, as well as decreases in overfitting and the reduction in computational costs [12].

We present a new hybrid AI framework, which combines transformer-based contextual language modeling with community-level graph reasoning as well as bio-inspired feature optimization for mental health surveillance on social media. First, a pipeline of SBO-based feature selection is used to better understand an assortment of linguistic and psychological features. We then pass those features through a fine-tuned BERT model and generate deep contextual embeddings. A Convolutional Neural Network (CNN) layer is employed with BERT outputs to better recognize localized high-risk expressions; this helps the model capture short, discriminative phrase patterns. The second is a GNN module that models the user–post interaction graph to learn relational patterns and detect high-risk clusters in the communities, as well as behavioral propagation. Key components within the framework are explainability and ethics compliance. Interpretability of both local and global predictions is explained using Explainable AI (XAI) methods such as SHapley Additive exPlanations (SHAP) and Local Interpretable Model-Agnostic Explanations (LIME). These methods not only highlight important tokens, phrases, and additional features on which the classification is based as a post-processing step (crucial for clinical validation and trust), but also sort them automatically. We test the suggested framework using two publicly available benchmark datasets that are commonly used in research of mental health NLP: eRisk [13] and CLPsych [14]. In addition, Reddit-based mental health classification has been widely explored in prior studies, including contextualized classification of Reddit posts using DSM-5 categories for web-based intervention [15]. The two datasets are both based on Reddit and are used to label risk indicators that are related to mental health with clinical labels. Although user-disjoint evaluation has been adopted as the standard evaluation protocol for early risk prediction (e.g., eRisk), this study adopts the same protocol for practical and reproducible generalization, rather than claiming originality.

This study is motivated by the need for multi-level mental health modeling in social media, and unlike previous research that mostly concentrates on either post-level classification or feature engineering, this paper focuses on a multi-level approach to model mental health in social media. Psychological signals are multi-faceted and include (i) linguistic and psychological features, (ii) contextual and local semantic expressions of each post, and (iii) user- and community-level relations. Current methods may focus on these separately, resulting in partial representations. To resolve this issue, we develop an integrated, multi-stage model in which each stage targets a specific problem in the modeling process: (i) feature optimization (SBO), (ii) semantic and local modeling (BERT + CNN), (iii) community and relational modeling (Graph Neural Networks), and (iv) explainability and trustworthiness for decision-making (SHAP/LIME). This approach allows for integrating different learning paradigms instead of simply integrating different techniques.

The key contributions of this paper are listed below:

➢ We design a multilevel text-based approach to model mental health status by incorporating feature-level, post-level, and community-level information for detecting depression and suicide.
➢ We propose a hybrid BERT-CNN representation module to integrate contextual semantic features and boost phrase-level risk pattern recognition in social media text.
➢ We investigate SBO-based bio-inspired feature selection to compact high-dimensional handcrafted linguistic and psychological features while retaining clinically relevant indicators.
➢ We combine GraphSAGE-based community reasoning, as well as explainability and calibration analysis with SHAP/LIME to enable interpretable, leakage-free, and reproducible evaluation with a user-disjoint protocol.

Overall, this paper attempts to fill the gap between individual mental health detection and community behavioral analysis on social media. Our proposed system, comprising state-of-the-art NLP and relational modeling, integrated with bio-inspired optimization in an explainable and ethically sound framework, provides a scalable, i.e., interpretable and robust solution to detect mental health crisis proactively. The remaining part of this paper is organized as follows: Section 2 discusses the related work on AI-based mental health detection, community, and feature optimization. Section 3: It is separated into three parts, i.e., proposed methodology, feature selection, and model architecture with community-level reasoning. Section 4: datasets, experimental setup, and evaluation measurement, provides the results and discussion of comparative and ablation studies. The last Section 5 wraps up our work and addresses future development.

## 2. Related Work

Initial research on mental health detection using social media was mainly based on conventional machine learning pipelines that involve the integration of manually engineered features and shallow classifiers. Sentiment through lexicon and linguistic characteristics combined with SVMs was applied to screening depression and Post-Traumatic Stress Disorder (PTSD) in short text [16], and behavioral/interaction alerts based on Reddit usage were investigated to predict the risk of postpartum depression [17]. Even though these strategies are interpretable and work well in limited contexts, they tend to miss context-based nuance, figurative language, and long-range dependencies where mental health disclosures often occur.

In order to address these shortcomings, there was a research transition to deep neural models in representation learning and text classification. CNNs, LSTMs, and hybrid RNN-CNN architectures enhanced performance by learning discriminative patterns directly from text [18]. This was further advanced by transformer-based models, which enable bidirectional context modeling and strong transfer learning; BERT-style encoders have been successfully used to detect suicidal ideation, depression, and stress, and generally outperform earlier baselines [19,20,21,22]. Later works explore domain-adaptive and mental-health-specific transformer variants to better capture conversational patterns [23,24], with comparative studies demonstrating the effectiveness of transformer encoders over recurrent models [25]. However, many transformer-based systems remain post-centric, focusing on individual posts or aggregated user summaries without explicitly modeling relational structures between posts and users.

Graph-based learning has been introduced to address this limitation by modeling relationships such as authorship, interactions, and semantic similarity through graph structures. Graph convolutional models have been used to analyze depression prevalence and identify community-level patterns [26], and temporal GNN extensions have been proposed to track changes in psychological states over time [27]. These studies demonstrate that graph-based modeling is already an established approach in mental health analysis, including explicit graph construction in several prior works. However, several practical challenges remain: (i) incomplete or inconsistent graph schema specification (e.g., unclear node and edge definitions), (ii) limited emphasis on controlled evaluation settings, particularly with respect to avoiding information leakage across user splits, and (iii) limited integration of graph reasoning with strong contextual encoders and feature optimization within a unified pipeline.

Another line of research focuses on improving efficiency and robustness through meta-heuristic optimization, particularly for high-dimensional feature selection. Systematic reviews show that such methods can reduce redundancy and improve generalization in text classification [28]. The Secretary Bird Optimization (SBO) algorithm has been introduced as a competitive global optimizer with an effective exploration–exploitation balance for selecting compact feature subsets [29]. However, in mental health NLP, bio-inspired optimization is often applied as a standalone preprocessing step or alongside shallow classifiers, with limited evidence of its integration with transformer-based representations in end-to-end frameworks.

In addition to predictive performance, transparency, reliability, and responsible deployment are critical for real-world mental health applications. Explainable AI techniques such as SHAP and LIME are widely used to interpret model predictions by highlighting influential features [30,31,32]. Ethical and governance-oriented research emphasizes privacy, risk mitigation, and responsible use in surveillance settings [33,34], while more recent studies address fairness and bias across heterogeneous populations [35]. Complementary directions include multimodal depression detection using speech and facial cues [36,37], longitudinal modeling of suicide risk patterns [38,39], and domain adaptation for cross-platform generalization [40,41]. Lightweight and real-time pipelines have also been proposed for crisis monitoring, highlighting the importance of deployable and uncertainty-aware systems [42,43].

In general, previous research has considered transformer-based modeling, graph-based reasoning, feature selection, and explainability as either independent or loosely interrelated components. Thus, this study has not contributed individually to the use of these techniques, but rather to their integration in a single and reproducible system. Although user-disjoint evaluation has been widely adopted for early risk prediction tasks, it is adopted here primarily for realistic generalization rather than as a contribution. In addition, although inductive GraphSAGE is used to enable generalization to unseen nodes, this study does not claim to directly compare the performance of this GNN variant with other alternatives, which is left as future work.

Table 1 summarizes prior studies, highlighting that existing approaches typically address contextual modeling, graph-based reasoning, and feature optimization independently.

## 3. Proposed Framework

**Figure 1:**
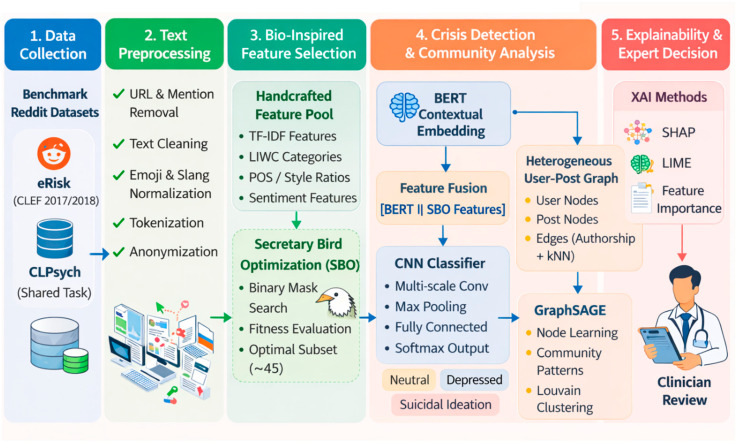
Overall architecture of the proposed hybrid framework combining SBO, BERT, CNN, GNN, and XAI for social media mental health surveillance.

The suggested framework offers a strong, interpretable, and community-conscious solution to identifying mental health crises and trends from social media content. It combines four main AI modules: (1) bio-inspired feature extraction, (2) transformer contextual embeddings, (3) CNN-augmented crisis identification, and (4) graph neural networks (GNNs) for inference at the community level, with a final Explainable AI (XAI) layer for ensuring interpretability and ethical transparency, as shown in Figure 1.

These modules are motivated by specific limitations of the existing approaches, rather than arbitrary design. Specifically, transformer-based models like BERT are well-suited to capture contextual semantics but lack a focus on local expressions of crisis that drive the use of CNN for phrase-level pattern extraction. Moreover, most current methods focus on the post level and do not model relational dependencies between users and posts; this is remedied with the use of an inductive Graph Neural Network (GraphSAGE) for user community-level inference. Moreover, manually designed psychological and linguistic features are informative but lead to a high-dimensional and redundant feature space, which is addressed through the Secretary Bird Optimization (SBO) algorithm for feature selection. Finally, due to the sensitive nature of applications in mental health, explainability and reliability are achieved via SHAP, LIME, and calibration analysis to ensure explainability, trust, and accountability. These modules combine to create a complementary and necessity-based architecture, rather than an amalgam of prior approaches.

The pipeline starts with a data acquisition module that ingests social media posts publicly available on platforms like Reddit and Twitter, which have keywords and metadata that are related to mental health. Various experiments in this study are all performed on released Reddit benchmark datasets (eRisk and CLPsych) only. Preprocessing is performed on the text before modeling, noise is removed, identifiers are anonymized, slang and emojis are normalized to standardize the text, making it robust to extract the features and downstream learning. To process the sparse, noisy, and high-dimensional inputs, Secretary Bird Optimization (SBO) is employed to select the most informative linguistic and behavioral features, including TF-IDF words, sentiment scores, part-of-speech ratios, and LIWC categories. This yields a small, high-quality feature set for deep learning. A finely tuned BERT model then projects all the posts into rich contextual embeddings. These embeddings are then passed through a Convolutional Neural Network (CNN), which extracts local phrase-level patterns and affective cues typical of depression or suicidal ideation. To generalize beyond per-post classification, a user–post interaction graph is learned by a GNN module where posts or users are nodes and interactions like replies, mentions, or co-occurring hashtags are edges. With GraphSAGE, the model detects clusters of high-risk and monitors potential escalation patterns within communities. Performance assessment is conducted through the utilization of precision, recall, F1-score, and ROC-AUC metrics on standardized datasets, including eRisk and CLPsych. To ensure transparency, SHAP and LIME methodologies emphasize significant tokens and features, thus facilitating interpretability for clinicians, moderators, and researchers. Overall, the method accomplishes three things: (1) correct high-risk content detection, (2) detection of community-level trends in mental health, and (3) ethically sound, interpretable decision-making.

## 4. Data Collection and Preprocessing

The suggested mental health surveillance construct is based on the prudent preparation of quality textual information that is acquired from open sources. The two fundamental elements of this step include (i) selection of the dataset and clear specification of tasks and label harmonization, and (ii) standardized preprocessing to make sure that the data sent to the next models are salient, consistent, and least influenced by platform noise.

### 4.1. Datasets and Ethical Use

The experiments are conducted using two publicly available Reddit-based benchmark datasets, eRisk and CLPsych Shared Task dataset. It is worth noting that the datasets are not synthetic, but these are standard benchmark datasets originally built from real Reddit data and distributed in research shared tasks (CLEF eRisk and CLPsych). The Kaggle links provided in this paper refer only to the mirror distributions of these datasets for ease of access, and do not represent a different (synthetic) data source. eRisk has user-level timelines that are curated to perform timely risk detection (i.e., depression-related disorders) on long user histories, and CLPsych includes posts annotated with suicide risk and related psychological issues using expert-determined protocols. The datasets are utilized as distributed by their terms; no effort is made to find, contact, or otherwise engage with any of the people depicted in the data.

#### 4.1.1. Harmonization of 3-Class Task Definition and Labels

Since eRisk and CLPsych were originally released under different shared-task formulations, we establish a unified 3-class label space consisting of Neutral, Depressed, and Suicidal Ideation to enable consistent evaluation across datasets. It is crucial to clarify that the annotations are not changed. The eRisk dataset includes only labels for depression risk, but not an explicit label for “Suicidal Ideation”. On the other hand, suicide-related labels are obtained from the CLPsych dataset only. As a consequence, the harmonization process introduces an alignment between different label spaces at the time of evaluation, without re-labeling the data. The claim “no re-labeling” means that we do not change the labels of the original datasets. The set of mapping rules used is shown in Table 2.

#### 4.1.2. Splits and Leakage Control of the Datasets (User-Disjoint)

To avoid leakage and provide a realistic generalization, we adhere to a strict user-disjoint splitting: posts of an individual user are in a single split (train/validation/test). This is especially significant to mental health NLP since the user-specific linguistic characteristics may artificially exaggerate performance in the post-level splitting. To be complete and reproducible, we have provided the user-level dataset statistics employed under the split protocol adopted, i.e., (i) the number of users in the dataset and each split, (ii) posts-per-user distribution (median/IQR) shown in Table 3.

#### 4.1.3. Text Preprocessing

**Figure 2:**
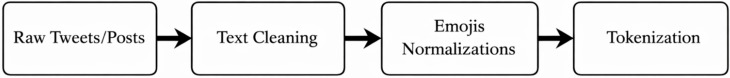
Preprocessing pipeline from raw tweets/posts to cleaned, normalized, and tokenized text for feature extraction.

Since social media text is noisy and conversational, a unified preprocessing pipeline is implemented on both datasets to minimize variance even though clinically meaningful cues are retained. Figure 2 shows that preprocessing involves URLs and user mentions removal, casing/whitespace normalization, token- level cleaning, elongated/stretched word normalization, and emoji/emoticon transliteration in order to retain the affective intent. Any identifying strings are encrypted in order to protect privacy. This standardized preprocessing enhances robustness and lowers consistency representations before feature extraction and modeling.

Finally, Table 4 gives an annotated sample of representatives in eRisk and CLPsych, which shows the variety of linguistic expressions and the homogenous mental health categories applied in this research.

### 4.2. Feature Selection Using Secretary Bird Optimization (SBO)

Textual indicators (TF-IDF, LIWC, POS-like ratios, sentiment cues) are handcrafted contextual embeddings supplementing contextual embeddings and depict explicit patterns of affect and style that can be utilized in mental health risk prediction. The full handcrafted pool, however, is high-dimensional and redundant, which adds risk of overfitting and computation to it. We thus use Secretary Bird Optimization (SBO) to use a small group of handcrafted features and retain predictive utility. The SBO algorithm is applied to enable joint optimization of diverse feature groups towards a single objective. Classical methods like L1-regularized linear regression (LASSO), recursive feature elimination (RFE), and mutual information-based methods are also viable alternatives, but SBO is used here as a general meta-heuristic that can learn non-linear interactions. This paper does not claim to be superior to all of these approaches, but provides an example of SBO as an optimization algorithm for the proposed framework. Table 5 lists the handcrafted feature pool size and SBO setting, with the original 5110-dimensional feature pool containing 5000 TF-IDF word n-gram features and 110 engineered linguistic and psychological features. The 110 engineered features consist of 93 features from LIWC, 12 POS/style ratio features and 5 sentiment features. SBO optimally reduces this 5110-dimensional feature pool to a subset of 45 features, which is used as final feature set in the proposed approach

Let the feature pool (crafted by hand) be F of total dimensionality N. All SBO candidate solutions are binary masks. The binary mask representation of each candidate solution is defined in Equation (1)

(1) $$z\in \{0,1{\}}^{N},S(z)=\left\{f_{j}\in F|z_{j}=1\right\},|S(z)|=\sum_{j=1}^{N}z_{j}.$$

The fitness function optimized by SBO is given in Equation (2), where classification performance is maximized while larger feature subsets are penalized:

(2) $$\mathrm{Fitness}(z)={\mathrm{Acc}}_{\mathrm{inner}\text{-}\mathrm{CV}\;}(S(z))-\lambda \cdot \frac{\left|S(z)\right|}{N}$$

Here, ${\mathrm{Acc}}_{\mathrm{inner}\text{-}\mathrm{CV}\;}(\cdot )$ is the mean accuracy from K-fold cross-validation within the training split, and λ penalizes overly large subsets. SBO’s fitness function uses inner cross-validation to ensure that no validation or test data are used for training. This approach reduces the possibility of overfitting and avoids data leakage between splits in the user-disjoint setting. After convergence (or reaching the iteration limit), SBO outputs $z^{*}$ and the selected subset $S^{†}$.

In our experiments, SBO selected 45 handcrafted features, whereas RF-importance retained 73, giving a 38.36 reduction in selected dimensionality. The percentage reduction in selected dimensionality relative to the RF-based baseline is computed using Equation (3):

(3) $$\mathrm{Reduction}\;=\frac{73-45}{73}\times 100\approx 38.36\%$$

The reduction in features is due to a compact and less redundant feature space; however, some features may still contain a weak signal predictive of suicide, and we will leave a detailed ablation study of features removed for future work.

All corpus-dependent statistics (TF-IDF vocabulary/IDF, scaling) are fitted on training data only and then applied unchanged to validation/test to prevent leakage. Some selected features are explicit lexical clues (e.g., suicide keywords), but the framework augments these with contextual and graph embeddings, making it less dependent on the presence of keywords for detection. The top-ranked selected features are reported in Table 6. We perform feature selection separately with the same protocol for each dataset. We do not presume that the selected features are transferable across datasets.

While some selected features represent explicit lexical cues (e.g., suicide-related words), the proposed model is not solely based on keyword matching. These explicit cues are integrated with contextual embeddings of BERT-CNN and graph-based representations, and reliance on lexical cues is reduced, leading to better contextualization

### 4.3. Contextual Embedding with BERT

For better mitigation of diverse syntactic and emotional aspects involved in targeted social media posts, the system utilizes BERT Bidirectional Encoder Representations from Transformers as its core contextual encoder by capturing co-related bidirectional attention to catch up with underlying subtle linguistic cues relevant to depression, anxiety, and suicide intention. Static embeddings (i.e., Word2Vec, GloVe) cannot capture contextual information and will not be able to make sense of phrases that are charged emotionally or written sarcastically in nature, unlike BERT which generates dynamic context-aware representations. The pre-trained BERT-base (12 layers, 768 hidden units, 12 attention heads) model is fine-tuned for domain adaptation on the mental health datasets–eRisk and CLPsych), which treats each post as an independent sample with every tokenized by the WordPiece tokenizer and prepended with a [CLS] token along with additional attention masks and segment embeddings. Where h[CLS] is the final hidden state of the token [CLS] which is used to represent/write down the entire sequence and then sent it to a Fully connected layer for classification. The post-level contextual representation generated from the [CLS] token is transformed for classification as expressed in Equation (4)

(4) $${\;\mathrm{Output}\;}_{\mathrm{post}\;}=\mathrm{Softmax}\left(W\cdot h_{\left[CLS\right]}+b\right)$$

Here W and b are learnable parameters. Optionally, optimal features from SBO (e.g., LIWC scores, POS ratios) can be concatenated with h[CLS], to have a fused vector. The fusion of the BERT contextual representation with SBO-selected handcrafted features is formulated in Equation (5)

(5) $$\mathrm{Fused}\_\mathrm{Vector}\;=\left[h_{\left[CLS\right]}\|\;\mathrm{SBO}\_\mathrm{Features}\;\right]$$

better exploit the complementarity of LIWC and POS, for example. These embeddings are then passed to the CNN layer for local pattern detection, as node features in GNN-based community analysis, and for Explainability through SHAP and LIME. Through this integration, the model learn at both the individual granular semantic level with specific clinical details and the community wide scale of behavior patterns important for mental health crisis detection.

### 4.4. Crisis Detection via BERT + CNN Hybrid

**Figure 3:**
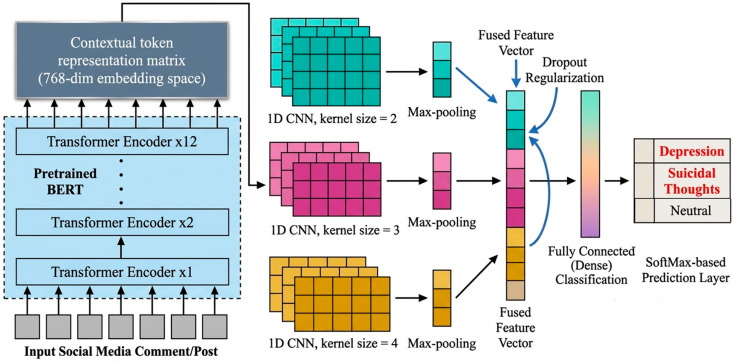
Architectural framework of the proposed hybrid BERT-CNN crisis detection model.

The proposed crisis detection module applies to a hybrid model that utilizes BERT and CNN to classify social media comments as depression, suicidal thoughts, or neutral (refer to Figure 3). BERT generates contextual embeddings for each token, $H=\left[h_{1},h_{2},\ldots ,h_{T}\right],h_{i}\in R^{768}$, thus capturing bidirectional semantics. BERT may capture long-range dependencies, but the CNN is added to capture local phrase-level dependencies (e.g., short n-grams like “I hate myself”) that are not explicitly highlighted by attention. To enhance localized, strong expression detection (i.e., “I hate myself”), the convolution operation used to extract local phrase-level patterns from contextual embeddings is defined in Equation (6).

(6) $$f_{i}=\mathrm{ReLU}\left({\mathrm{Conv}}_{i}(H)\right)$$

Each feature map is max-pooled to preserve the strongest activation, concatenated, and dropout regularized. The pooled and regularized feature representation obtained after convolution is expressed in Equation (7).

(7) $$p_{i}=max\left(f_{i}\right)$$

The final class probability computation through the fully connected SoftMax layer is given in Equation (8).

(8) $$\mathrm{Output}\;=\mathrm{Softmax}\left(W\cdot \left[p_{1}\left\|p_{2}\right\|\ldots \|p_{k}\right]+b\right)$$

The model is cross-entropy loss trained with class weighting or oversampling for imbalance. Predicted labels and output activations are fed to the GNN module for community reasoning and the XAI module for interpretability. This architecture bridges BERT’s semantic depth and CNN’s phrase-level accuracy to support robust detection of mental health crises. The training objective of the BERT-CNN classifier is defined by the cross-entropy loss in Equation (9).

(9) $$L=-\sum_{c=1}^{C}y_{c}\log\left({\hat{y}}_{c}\right)\;$$

The output of the BERT-CNN module serves as the primary post-level prediction, which is subsequently used as input to the GNN for relational refinement and community-level reasoning. An ablation comparing other fusion strategies or models (e.g., without CNN, different fusion schemes) is out of the scope of this paper and is left for future work.

### 4.5. Community-Level Graph Reasoning with Graph Neural Networks

#### 4.5.1. Task Definition and Learning Objective

The Graph Neural Network (GNN) has two somewhat different uses: (1) supervised mental health risk classification, and (2) unsupervised post hoc community analysis. In the proposed approach, the majority of the classification is performed by the BERT-CNN module, and the GNN module is used to supplement it by learning from the graph structure. During the supervised training, the GNN is used to classify post nodes’ mental health risk labels using gold-standard labels, primarily for learning the graph-aware features, rather than primary classification. The learned representations are used for community detection with no labels involved, to understand the risk structure at the community level. No labels are used for clustering, and the clustering process does not interfere with the supervised training. Therefore, the predictions are provided by BERT-CNN, and are refined by GNN based on community-level knowledge

#### 4.5.2. Graph Construction and Schema

We build a heterogeneous user post graph G (V, E), with the node set V consisting of two classes of nodes: user and post nodes. Edges encode (i) authorship links connecting each post node to its author (user node) and (ii) semantic similarity links between posts. Cosine similarity between Bert + CNN representations is used to weigh semantic edges, and where applicable, interaction edges are weighted with normalized interaction frequency. Edges are all made undirected to indicate the reciprocal flow of information, and self-loops are introduced to retain the node-specific information in passing along the messages.

***Semantic edge formation***. For each post node $p_{i}$, we compute cosine similarity $s_{ij}=\cos\left(z_{i},z_{j}\right)$ between its BERT + CNN representation z and all other post embeddings within the same split. To prevent any leakage, the BERT + CNN model for generating graph-construction embeddings is trained on the training set and then frozen for producing validation and test embeddings. As such, the edges of the validation/test graph are determined using frozen representations without access to validation/test labels. We then connect $p_{i}$ to its top k nearest post neighbors (kNN) to form semantic edges. We tune (k,τ) on the validation set and select k=15 and τ=0.60, which yields a well-connected yet noise-controlled graph. Accordingly, an edge (i,j) is retained only if $s_{ij}\geq 0.60$; otherwise, it is discarded. If fewer than k neighbors satisfy the threshold, we keep all available neighbors above τ, resulting in a degree <k for that node. The semantic graph is symmetrized (undirected) by taking the union of KNN edges, and edge weights are set to $w_{ij}=s_{ij}$. For stable message passing in GraphSAGE, we apply symmetric normalization to the weighted adjacency with self-loops, $\tilde{A}=D^{-1/2}(A+I)D^{-1/2}$, where D is the degree matrix and I add self-connections.

To clarify, cosine similarity is only used to determine initial graph structure and weights, and is not used for relational learning. The GNN is not redundant because GraphSAGE learns task-specific features for each node by aggregating its neighborhood over this graph structure. Unlike traditional pairwise similarity, GraphSAGE supports multi-hop neighborhood aggregation, so each post can propagate (and learn) from its related posts and users. Hence, the GNN provides community-scale relational reasoning on top of the semantic similarity-based graph construction.

#### 4.5.3. Node Features and Message Passing

The nodes are set to be initially represented by the BERT-CNN representation, instead of the raw BERT embedding. The BERT representation captures contextual embeddings of a post, and the CNN learn fine-grained risk patterns from phrases. The BERT-CNN vector is used as the primary node feature vector for each post node in GraphSAGE. Linguistic and psychological features (TF-IDF, LIWC, sentiment, POS-based features) selected by SBO are concatenated as supplemental interpretable features. Aggregated user statistics can also be used. Output logits or class probabilities of BERT-CNN are only used as optional confidence-aware features and not as labels/pseudo-labels or supervision for GNN training. The GraphSAGE-based neighborhood aggregation and node representation update are defined in Equation (10).

(10) $$h_{v}^{\left(l+1\right)}=\sigma \left(W^{\left(l\right)}\cdot \mathrm{AGG}\left(\left\{h_{u}^{\left(l\right)}:u\in N(v)\right\}\cup \left\{h_{v}^{\left(l\right)}\right\}\right)\right)$$

where $h_{v}^{\left(l\right)}$ denotes the embedding of node v at layer l, N(v) represents the set of neighboring nodes of v, AGG refers to the aggregation function used to combine neighborhood information, such as mean, max, or attention-based aggregation, and σ denotes a non-linear activation function, such as ReLU.

#### 4.5.4. Supervised Training and Loss Function (Ground Truth Only)

The GNN is estimated by the cross-entropy loss based on labeled post nodes, with the labels being considered only based on the ground truth annotations of the benchmark datasets. Notably, no post-level predicted labels are used as training targets (i.e., no pseudo-label supervision is used); the model is trained only on labels of the ground truth dataset. Class-weighted cross-entropy is used in the supervised training to fix the issue of class imbalance, particularly in the case of suicidal ideation. It only uses supervision in the classification objective, but not in the community detection.

#### 4.5.5. Community Detection and Analysis (Post Hoc, Unsupervised)

Upon training, the final-layer node embeddings are found and used with an unsupervised community detection algorithm, namely Louvain modularity optimization. This stage groups users and posts with similar learned representations, thus allowing qualitative and quantitative examination of risk structure at the community level. Community detection is thus applied only to analysis and does not interfere with the learnt classifier parameters.

#### 4.5.6. Leakage Control and Evaluation Protocol

Training and message passing in a selected training split are limited to avoid information leakage. The graph of training is only built with training nodes and edges, and the GNN is optimized only with ground truth labels of training post nodes only; validation and test nodes (and their labels) are never used during training. It is embedded at inference time, by validation/test nodes, using the learned GraphSAGE aggregation function (inductive inference). In these evaluation nodes, the connection between two points is established based on a fixed, label-free semantic rule (e.g., top (k) nearest neighbors of text-based neighbors taking out BERT + CNN representations under cosine similarity), which enables graph connectivity to be generated using only the attributes of the text between pairs, not the labels. The embeddings derived on the evaluation split are then subjected to community detection, which does not interfere with the supervised classifier parameters and does not induce any bias in the reported classification results.

We use the same fixed graph construction hyperparameter (k = 15, τ = 0.60) across splits. Importantly, kNN retrieval and thresholding are performed within each split only (train graph from train nodes; test graph from test nodes) and the normalized adjacency, $\tilde{A}=D^{-1/2}(A+I)D^{-1/2}$ is computed per split, preventing cross-split edges and information leakage.

### 4.6. Model Evaluation Metrics

In order to evaluate the performance of the model, the important performance parameters, i.e., accuracy, precision, recall, F1-score and AUC were calculated using the following Equations

(11) $$\mathrm{Accuracy}\;=\frac{TP+TN}{TP+TN+FP+FN}$$

(12) $$\mathrm{Precision}\;=\frac{TP}{TP+FP}$$

(13) $$\mathrm{Recall}\;=\frac{TP}{TP+FN}$$

(14) $$F1=2\times \frac{\;\mathrm{Precision}\;\times \;\mathrm{Recall}\;}{\;\mathrm{Precision}\;+\;\mathrm{Recall}\;}$$

(15) $$\mathrm{AUC}=\int_{0}^{1}\mathrm{TPR}(FPR)dFPR$$

Community-level metrics, Modularity Score (cluster quality), Node Classification Accuracy and Silhouette Coefficient (cluster compactness/separability) are used for GNN Outputs. Rigorous evaluation based on multiple metrics ensures that the proposed method can achieve reliable detection, robustness under imbalance, and capture both individual and community-level mental health patterns.

### 4.7. Explainability and Ethical Considerations

Our proposed framework includes explanations and epistemic safeguards to promote transparency, trustworthiness, and responsible conduct in carrying out mental health surveillance. This article introduces Explainable AI techniques, based on SHAP (compute Shapley value for feature-specific word/phrase/SBO contribution to the prediction) and LIME (local surrogate model to characterize key terms for each classification), that are integrated to generate explanations from BERT + CNN/GNN module outputs at the global level as well as the instance level.

**Figure 4:**
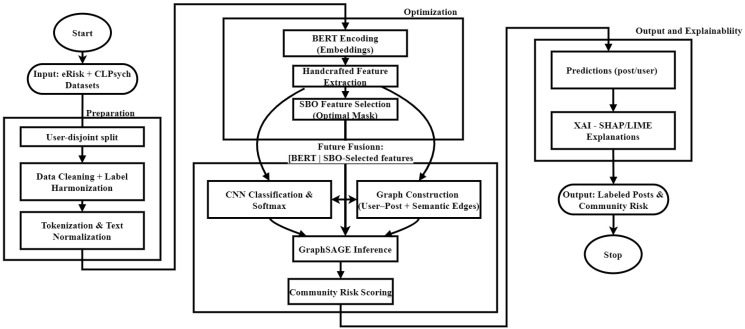
Flowchart of the proposed MENTAL pipeline for explainable crisis detection.

Maintained Ethical Compliance (Privacy & anonymity, publicly available data, PII Stripped, Dataset Licenses adherence; IRB Guidelines; Reducing Demographic Skew using Bias Mitigation Strategies). This high-risk prediction is not diagnostic and should be reviewed by a clinician before a planned intervention. Thus, the system consolidates interpretability, privacy-preserving and bias-aware practices for robust, fair and ethical human-centered deployment in sensitive mental health applications. To explain the process of operation of the proposed methodology, the full MENTAL_PIPELINE of transparent detection of crisis, with the description of each part of it, can be found in Algorithm 1 and Figure 4, which starts with the benchmark data acquisition and processing and continues through SBO-directed feature selection, post-level classification with BERT and CNN, and community-level reasoning provided by GNNS. The pipeline also introduces XAI-based interpretability (e.g., SHAP/LIME) to interpret model decisions, thus delivering a structured and step-by-step end-to-end structure that aids in both precise prediction and responsible analysis.
**Algorithm 1: Explainable Crisis Detection via SBO-BERT-CNN with Inductive GraphSAGE Refinement****Input**: Labeled posts $D={\left\{\left(x_{i},u_{i},y_{i}\right)\right\}}_{i=1}^{N}$; label map M; preprocessing Clean(⋅) and tokenizer Tok(•); handcrafted extractor ϕ(⋅) with dimension d; SBO settings $\left(N_{p},T_{\max\;},m\right)$; BERT encoder $E_{\theta }$; CNN head $C_{\psi }$; class weights ${\left\{w_{k}\right\}}_{k=1}^{K}$ with K=3; graph params $\left(k,\tau ,L_{g}\right)$; GraphSAGE parameters ω; Louvain; SHAP/LIME.
**Output**: Post predictions $\left({\overset{ˆ}{y}}_{i},p_{i}\right)$, optional graph-refined $\left({\overset{ˆ}{y}}_{i}^{g},p_{i}^{g}\right)$; communities and risk scores $r_{c}$; explanations $E_{i},E_{\mathrm{global}\;}$
  1.**Harmonize labels**: $y_{i}\leftarrow M\left(y_{i}\right)$. Create user-disjoint splits $U_{tr},U_{va},U_{te}$ and datasets $D_{s}=\left\{\left(x_{i},u_{i},y_{i}\right):u_{i}\in U_{s}\right\}$.  2.**Preprocess + represent**: For each $\left(x_{i},u_{i},y_{i}\right)$, compute ${\tilde{x}}_{i}=\mathrm{Clean}\left(x_{i}\right)$,  $t_{i}=\mathrm{Tok}\left({\tilde{x}}_{i}\right)$, and handcrafted features $f_{i}=\phi \left({\tilde{x}}_{i}\right)\in R^{d}$. Normalize $f_{i}$ using train statistics only.  3.**SBO feature selection**: Use a binary mask $z\in \{0,1{\}}^{d}$ with $\|z{\|}_{0}=m$ and selected features $f_{i}^{⋆}=f_{i}$

               
$z^{⋆}=\arg\underset{z,\|z{\|}_{0}=m}{\max}\mathrm{Macro}F1_{va}\left(M_{cls}(z)\right),$

           then fix $f_{i}^{⋆}=f_{i}⊙z^{⋆}$.
  4.**BERT fusion embedding**: Compute $H_{i}=E_{\theta }\left(t_{i}\right),h_{i}=H_{i}[$ CLS ], and fuse
                     
$s_{i}=\left[h_{i}\|f_{i}^{⋆}\right]\in R^{p+m}$
  5.**Post-level CNN** classification: Obtain logits $o_{i}=C_{\psi }\left(s_{i}\right)$,
     Probabilities $p_{i}=\mathrm{softmax}\left(o_{i}\right)$, and ${\hat{y}}_{i}=\arg\underset{k}{\max}p_{i}[k]$. Train by minimizing

                $L_{cls}(\theta ,\psi )=-\sum_{i\in D_{tr}}\sum_{k=1}^{K}w_{k}1\left(y_{i}=k\right)\log\;p_{i}[k].$
  6.**Leakage-free graph construction**: For each split s∈{tr,va,te}, build an isolated heterogeneous graph $G_{s}=\left(V_{s},E_{s}\right)$ with nodes $V_{s}=P_{s}\cup U_{s}$. Add authorship edges $\left(u_{i},p_{i}\right)$. Add semantic post-post edges using cosine similarity
                      $\mathrm{sim}(i,j)=\frac{s_{i}^{⊤}s_{j}}{\left\|s_{i}\right\|\left\|s_{j}\right\|},$
  connecting each post to its k nearest neighbors and keeping edges with sim(i,j)≥τ. Initialize $x_{p_{i}}^{\left(0\right)}=s_{i}$ and $x_{u}^{\left(0\right)}=\mathrm{mean}\left\{x_{p}^{\left(0\right)}:p\in N(u)\right\}$.
  7.**GraphSAGE refinement (inductive)**: For layer l,
             $m_{v}^{\left(l\right)}=\mathrm{AGG}\left\{x_{u}^{\left(l\right)}:u\in N(v)\right\},x_{v}^{\left(l+1\right)}=\sigma \left(W^{\left(l\right)}\left[x_{v}^{\left(l\right)}\|m_{v}^{\left(l\right)}\right]\right).$
      For each post $i_{,\;\mathrm{set}\;}g_{i}=x_{p_{i}}^{\left(L_{g}\right)},p_{i}^{g}=\mathrm{softmax}\left(W_{g}g_{i}\right),{\hat{y}}_{i}^{g}=\arg\underset{k}{\max}p_{i}^{g}[k]$.
      Train on $G_{tr}$ with
               
$L_{gnn}(\omega )=-\sum_{i\in D_{tr}}\sum_{k=1}^{K}w_{k}1\left(y_{i}=k\right)\log\;p_{i}^{g}[k],$
       then apply inductively to $G_{va},G_{te}$.
  8.**Community risk scoring**: Run Louvain to obtain communities c. For each community,
                   $r_{c}=\frac{1}{\left|P_{c}\right|}\sum_{p_{i}\in P_{c}}p_{i}^{g}[\;\mathrm{Suicidal}\;]$
  9.**Explainability**: Compute global SHAP summary $E_{\mathrm{global}\;}$ and per-post local explanations $E_{i}$ using LIME/SHAP for tokens and SBO-selected features.
      Return: $\left\{\left({\hat{y}}_{i},p_{i}\right)\right\}$, optional $\left\{\left({\hat{y}}_{i}^{g},p_{i}^{g}\right)\right\},\left\{r_{c}\right\}$, and explanations $\left\{E_{i}\right\},E_{\mathrm{global}\;}$

## 5. Results and Discussion

### 5.1. Overall Performance of the Proposed Framework

The hybrid framework is evaluated on eRisk benchmark dataset and CLPsych Shared Task dataset for identifying neutral, depressive and suicidal ideation social media posts. Under a strict user-disjoint evaluation protocol, the full SBO + BERT + CNN + GNN pipeline achieved the best overall performance among all compared methods, with an accuracy of 93.1%, F1-score of 0.91, and ROC-AUC of 0.944. These results indicate that the proposed framework provides robust and reliable detection of high-risk mental health content across benchmark datasets.

**Figure 5:**
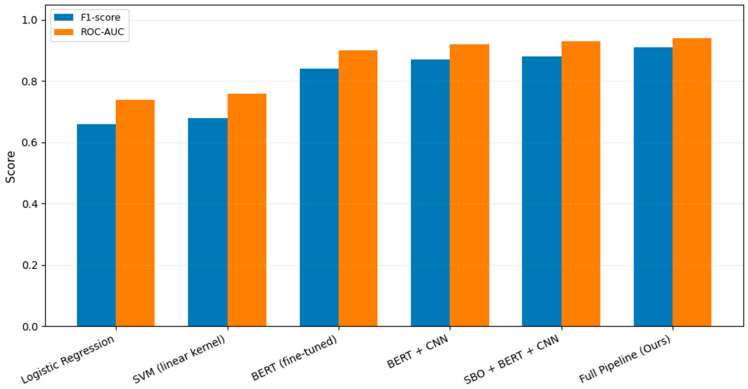
Bar chart comparison of F1-score and ROC-AUC across different models.

The suggested SBO + BERT + CNN + GNN pipeline was compared with classical classifiers (Logistic Regression, SVM) and deep learning baselines (BERT, BERT + CNN, SBO + BERT + CNN) on accuracy, precision, recall, F1-score, and ROC-AUC. As can be seen in Table 7 and Figure 5, classical models performed well on depressed and neutral classes but poorly on suicidal ideation. Fine-tuned BERT enhanced performance through contextual embeddings, and the addition of CNN enhanced recall and F1-score through local n-gram pattern capture. The addition of SBO enhanced feature dimensionality reduction without sacrificing accuracy, and GNN enhanced community-level risk detection through relational signals from user post graphs. The end-to-end pipeline had the best performance (Accuracy = 93.1%, F1 = 0.91, ROC-AUC = 0.944), indicating robustness in identifying high-risk mental health content.

### 5.2. User-Level Risk Aggregation Results

**Figure 6:**
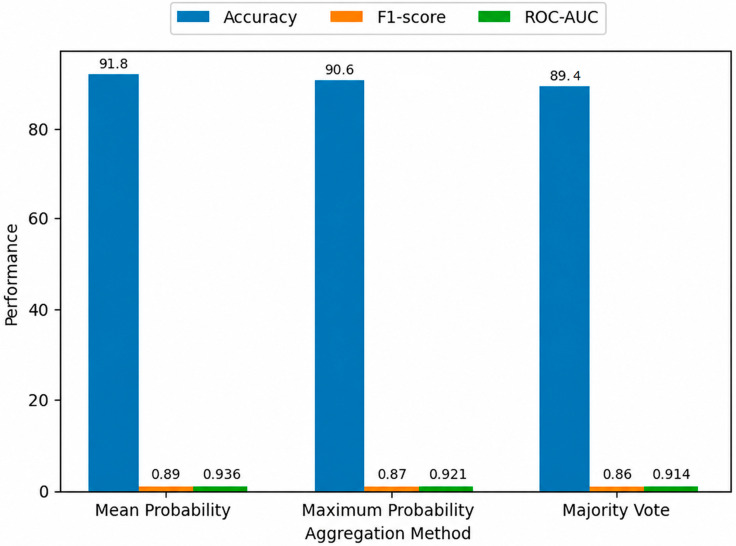
User-level mental health risk detection performance using different aggregation strategies applied to post-level predictions. Numerical values are shown on bars for accuracy (%), F1-score, and ROC-AUC.

Mental health assessment is inherently user-friendly, although the model evaluation is carried on the post level. To test the hypothesis of post-level prediction being a reliable user-level risk predictor, we conducted a user-level aggregation analysis of both eRisk and CLPsych test splits using the current model predictions (not retrained). Post-level predictive probabilities were aggregated using three strategies, mean probability, maximum probability, and majority voting and the strongest user-level performance was obtained with mean probability aggregation (Accuracy = 91.8%, F1-score = 0.89, ROC-AUC = 0.936), as summarized in Table 8. Maximum probability aggregation has competitive performance, but it is more prone to local high-risk posts, whereas majority voting performs poorly in the case of risk signals that are subtle or expressed intermittently across posts. The relative dynamics among the aggregation strategies are further demonstrated in Figure 6 that indicates that the aspect of mean-based aggregation is more stable compared to the other methodologies. These findings suggest that the suggested framework generates coherent post-level predictions that can be successfully aggregated to useful user-level predictions of risk. Notably, the analysis is not a temporal early-risk detection analysis; instead, it indicates consistency between post-level and user-level analysis in an offline evaluation environment.

To ensure that user-level aggregation does not impact performance, user aggregation was performed only among the held-out test users using their post-level prediction probabilities. We did not use any posts from training/validation users. We also compared performance on users with different activity patterns, dividing users into low-activity users (1–2 posts), moderate-activity users (3–10 posts), and high-activity users (>10 posts), to see whether aggregation performance is consistent across varying distributions of post counts.

Besides the aggregated comparison in Table 8 and Figure 5, we also provide the per-dataset post-level results of eRisk and CLPsych to make sure that the overall gains are not based on one benchmark. As Table 9 demonstrates, the proposed full pipeline obtains generally high accuracy, F1-score and ROC-AUC on both datasets, which confirms their cross-dataset robustness when using the same evaluation protocol of user-disjointing. 

### 5.3. Class-Wise Performance and Error Characterization

Although general measures allow a general comparison on a global scale, mental health risk detection needs a class-sensitive assessment, especially when it comes to suicidal thoughts, which are usually a minority and the most at risk group. Accordingly, we also provide class-wise precision, recall, and F1-score of the suggested full pipeline in Table 10 to confirm that the excellent overall results are not due to the majority neutral class alone.

As can be seen in Table 10, the model has good results in all the classes and high recall in suicidal ideation, which is particularly relevant in screening-based contexts where conservative false alarms that are conservative are less damaging than missed high-risk posts. The model design gains belong to the class-wise gains: BERT is sensitive to contextual distress, CNN is sensitive to the identification of short spans on crisis-related situations, and SBO is sensitive to reducing the redundancy of noisy features that can hinder minority-class discrimination.

**Figure 7:**
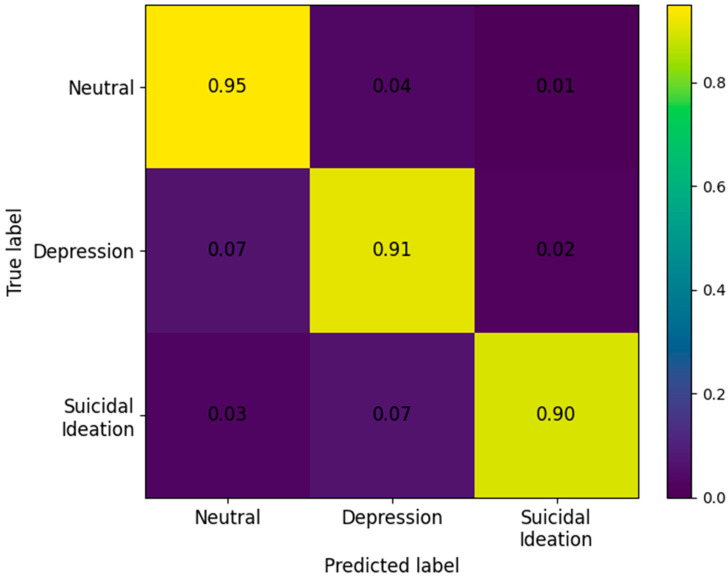
Normalized confusion matrix of the proposed full pipeline (Ours) on the test set (row-normalized).

To examine the patterns of errors further, Figure 7 provides the normalized confusion matrix of the proposed full pipeline. According to the matrix, the residual errors are concentrated on the depression and suicidal ideation, which depicts linguistic overlap in the distress stories (e.g., hopelessness vs. explicit self-harm intent). The generalizability of suicidal ideation to depression is still under-represented, indicating that the model is sensitive to the explicit crisis manifestations but not the generalized depressive manifestations. This error structure assists in interpreting that the suggested framework implements a balanced post-level risk identification, and on top of this, community-level graph analysis can be followed on a higher-level monitoring without suggesting the existence of the temporal early-risk definition.

As shown in Table 11**,** the proposed full pipeline remains consistent over five random seeds, achieving a Macro-F1 of 0.910 ± 0.006 and a suicidal recall of 0.900 ± 0.011. The low standard deviations indicate that performance is stable across runs and not driven by a favorable initialization, supporting reliable screening of high-risk posts.

To endorse the idea of triage-centered interpretation, we present an analysis of the operating point of a suicidal risk screening at different decisions as shown in Table 12. The decrease in the threshold causes suicidal recall to be higher (e.g., t = 0.30) but the workload of false alarms to increase, whereas the increase in threshold decreases the false alarms (e.g., t = 0.70) at the expense of false misses of high-risk posts. Mid-range threshold (t = 0.50) offers a realistic balance, both being highly recall with a low number of false alarms per 1000 posts which, together with the confusion matrix insights in Figure 7, serve to complement the insights offered by the confusion matrixes.

### 5.4. Ablation Study

**Figure 8:**
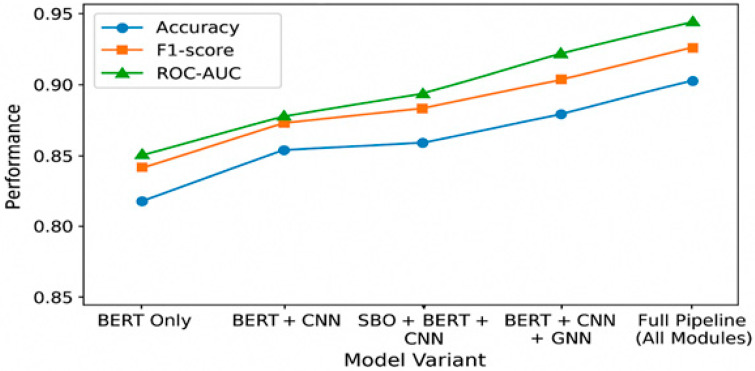
Line graph illustrating performance degradation in ablated models.

An ablation study was performed to analyze the contribution of each module—SBO, CNN, and GNN—within the proposed pipeline. Table 13 presents a progressive component-wise analysis rather than a strict leave-one-component-out ablation, while Figure 8 provides the corresponding visual comparison. The BERT-only baseline achieves strong contextual performance but lacks robustness in subtle and edge-case expressions. Adding CNN improves detection of localized crisis phrases, while incorporating GNN further enhances performance through community-level relational reasoning. The slight variation observed between SBO + BERT + CNN (91.2%) and BERT + CNN + GNN (91.4%) indicates that feature selection and relational modeling contribute differently and are complementary rather than independently optimal. Without SBO, redundant high-dimensional features reduce efficiency and slightly degrade performance. Without CNN, the model loses localized phrase detection capability. Without GNN, community-level reasoning is absent, limiting detection of socially clustered distress. The end-to-end pipeline achieves the best overall performance (Accuracy = 93.1%, F1 = 0.91, ROC-AUC = 0.944), demonstrating that the combined use of feature optimization, contextual modeling, and relational reasoning provides complementary benefits. A systematic ablation where each component (SBO, CNN, and GNN) is individually removed from the full pipeline is left for future work.

### 5.5. Calibration, Uncertainty, and Cross-Dataset Robustness

**Figure 9:**
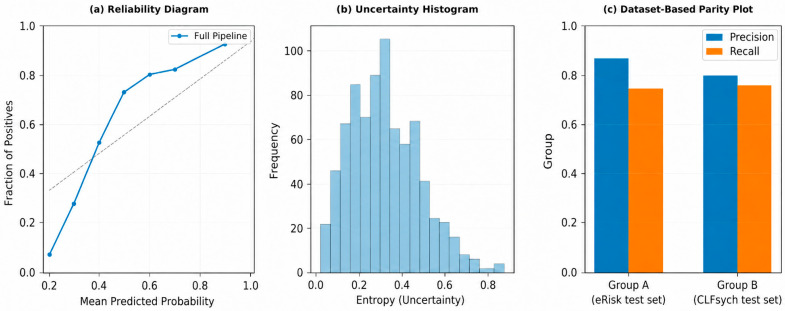
Full-pipeline calibration and uncertainty: (**a**) reliability diagram, (**b**) predictive entropy histogram, and (**c**) precision–recall comparison for eRisk and CLPsych.

To assess model reliability beyond standard classification metrics, we analyze calibration, predictive uncertainty, and cross-dataset robustness (precision/recall consistency across the eRisk and CLPsych test sets), as summarized in Figure 9. The reliability diagram in Figure 9a compares predicted confidence with empirical correctness We further evaluate how well the proposed model is calibrated using the Expected Calibration Error (ECE) and Brier score, as in Equation (16). Our model achieves **ECE = 0.048** and **Brier = 0.092** on the eRisk dataset, and **ECE = 0.052** and **Brier = 0.097** on the CLPsych dataset, suggesting stable model calibration across both datasets.

(16) $$\mathrm{ECE}=\sum_{m=1}^{M}\frac{\left|B_{m}\right|}{n}\left|\mathrm{acc}\left(B_{m}\right)-\mathrm{conf}\left(B_{m}\right)\right|,\;\mathrm{Brier}\;=\frac{1}{n}\sum_{i=1}^{n}{\left(p_{i}-y_{i}\right)}^{2}$$

Predictive uncertainty is reported in Figure 9b using entropy, the entropy-based uncertainty measure used to quantify predictive ambiguity is given in Equation (17)

(17) $$H(p)=-\sum_{c=1}^{C}p_{c}\log\;p_{c}$$

The higher the entropy, the more uncertain the prediction, which can be sent for human review in practice using uncertainty thresholds, though this is not explicitly defined in this work. Finally, Figure 9c compares performance across two dataset-defined groups, Group A (eRisk test set) and Group B (CLPsych test set), to evaluate cross-dataset robustness (no demographic attributes are inferred). Precision and recall show only minor disparity between groups (Group A: Precision = 0.86, Recall = 0.67; Group B: Precision = 0.85, Recall = 0.68), indicating stable performance across the two benchmark sources.

### 5.6. Feature Selection Effectiveness

Text classification often involves high-dimensional, sparse feature spaces even in the mental health domain; feature selection is crucial to our study to improve generalizability, reduce computational cost and enhance interpretability. We evaluate our SBO approach with RF importance using a collection of 1- and 2-tf-idf, LIWC psychological traits, VADER sentiment scores, and metadata. This has been done to achieve the highest classification accuracy (especially for suicidal ideation) while keeping the selected feature subset as compact as possible, as defined in Equation (2).

**Figure 10:**
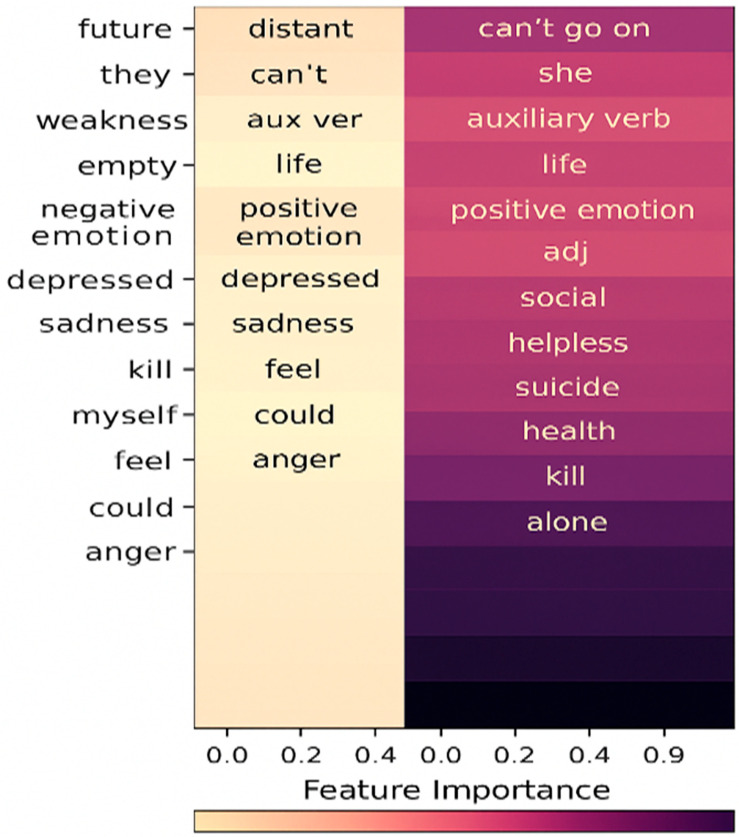
Feature importance heatmap from SBO-selected features.

As observed in Table 14, 45 features were chosen by SBO (≈38% less than RF), producing better accuracy (91.2%), F1-score (0.88), and ROC-AUC (0.926) which proves superior generalization and prediction capabilities for crisis detection. Random Forest importance is used as a reference baseline for comparison. A comprehensive evaluation against additional feature selection methods, such as LASSO, RFE, and Boruta, is beyond the scope of this study and is identified as future work. The feature importance heatmap. Figure 10 highlights emotionally and psychologically loaded language (e.g., “die”, “hopeless”, “sadness”. “self-focus”), verifying that SBO can keep semantically and clinically meaningful signals, while excluding redundancy noise.

These results further reinforce SBO as a concise, explainable and effective feature selector, allowing its insertion prior to context modeling in our pipeline. Feature selection is performed independently for each dataset under the same protocol. Therefore, cross-dataset transferability of selected features is not assumed in this study and remains an open direction for future investigation.

### 5.7. Feature Relationship Analysis

**Figure 11:**
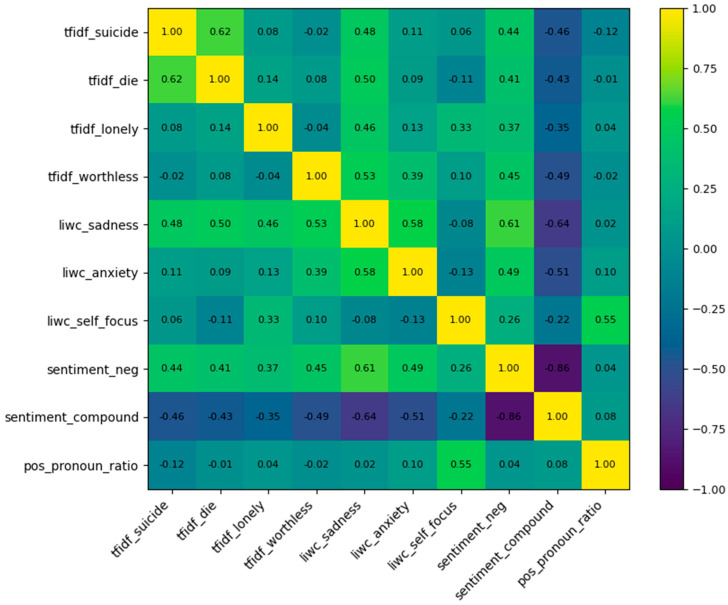
Correlation heatmap of selected linguistic and psychological features, showing positive co-variation among related risk cues and negative associations reflecting opposing sentiment polarity.

To evaluate the redundancy and dependency structure of the linguistic and psychological signals that the proposed framework takes advantage of, we calculate pairwise correlations of the representative features of SBO-selected features and plot them in the matrix in Figure 11. The correlation structure suggests interpretable groupings based on (i) crisis lexical indicators (TFIDF words), (ii) affective and psycholinguistic indicators (e.g., LIWC sadness/anxiety), and (iii) sentiment-induced indicators. As anticipated, there are positive associations between affective distress indicators and negative-sentiment elements, and negative-sentiment measures and compound polarity, and the overall directional behavior of affective distress and sentiment orientation is consistent. All this analysis suggests the importance of optimization of the SBO-based features: a number of handcrafted cues encode the same information, and the choice of a small set of them can be used to reduce redundancy and preserve salient discriminative features, which, in turn, can enhance robustness and generalization to noisy social media text.

### 5.8. Community Detection Insights

**Figure 12:**
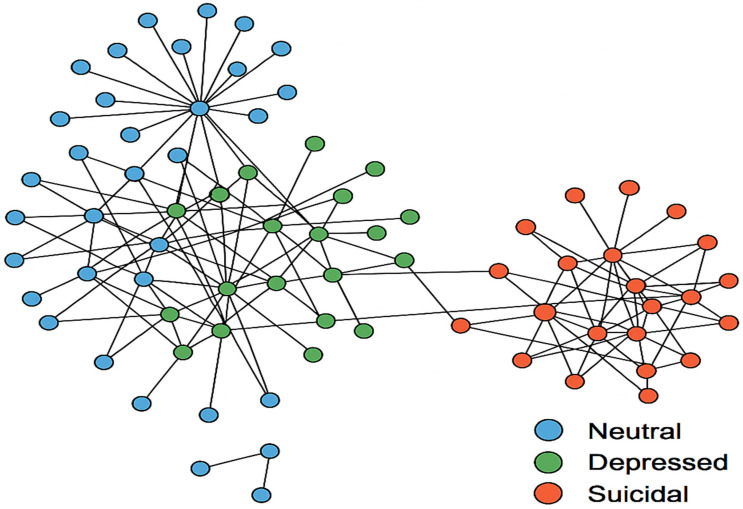
GNN-detected communities of at-risk users based on post similarity and interaction.

Apart from the post-level classifier, the model utilizes a GraphSAGE-based GNN to explore community-level risk patterns based on a user–post graph constructed from authorship links and semantic similarity between posts. This analysis is an exploratory post hoc examination of relational structure rather than an evidence of risk propagation. Figure 12 shows a qualitative view of the community structure. As illustrated in Figure 12, suicidal risk nodes appear to form relatively dense clusters in the constructed graph, while some depressive risk nodes appear near similar regions. But this is interpreted qualitatively and not as causal evidence of escalation or propagation. The GNN attained 91.4% accuracy in post-node classification on the held-out test split. The learned embeddings also produced separable community patterns, with average internal edge density of 0.61 and Louvain modularity of 0.73, as summarized in Table 15.

The detected modularity is affected by the kNN graph construction, and is therefore reported as an exploratory analysis. Comparisons with null models (e.g., degree-preserving random graphs) and quantitative examination of the within- versus between-community connections are left for future work.

### 5.9. Explainability Analysis

**Figure 13:**
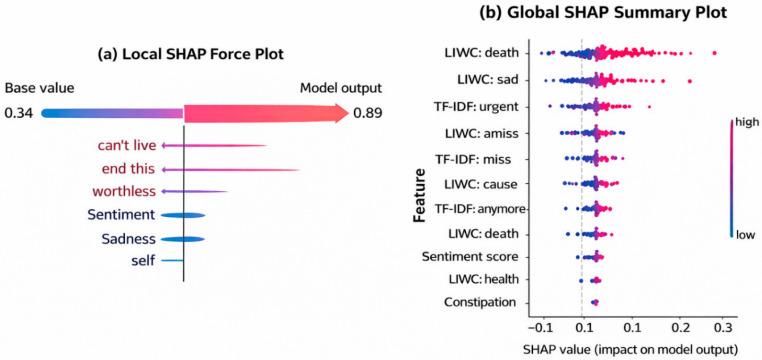
SHAP explainability results showing (**a**) local feature contributions for a suicidal post and (**b**) global feature importance across the test set.

Explainability is important beyond conventional accuracy metrics for deploying mental health surveillance models in sensitive, high-stakes environments. We used SHAP to interpret predictions locally (on a per-post basis) as well as globally within the feature space, aiding transparency in identifying depression and suicidal ideation. For consistency, KernelSHAP was used with 100 background samples drawn from the training set. SHAP values were calculated using the BERT-CNN output, and SBO-selected features, with LIME used at the post level using locally weighted surrogate explanations. Kernel SHAP: The predictions from the BERT + CNN classifier and features selected by SBO (TF-IDF, LIWC, sentiment) were input into KernelSHAP to assess the importance of each feature in determining prediction outcomes. At the local level, as shown in Figure 13a, the SHAP force plot reveals that lexical and linguistic properties contribute to suicidal risk prediction. At the global level, the SHAP summary plot in Figure 13b shows that LIWC categories (e.g., death, sadness), crisis-related terms, and sentiment scores are key separators across the dataset. These results indicate that the model’s decision-making aligns with psychologically relevant indicators.

Table 16 ranks the ten most influential features, with “die” (TF-IDF), sadness (LIWC), and negative polarity scoring highest in mean SHAP contribution. While direct cues such as “die” and “end it” feature prominently among the top features, the model does not solely rely on explicit keywords. For instance, a post like “I feel like I am slowly disappearing, and nothing feels real anymore” was correctly classified as suicidal ideation, yet does not contain explicit suicide keywords, where the explanation showed indirect cues of distress such as emptiness and hopelessness.

**Figure 14:**
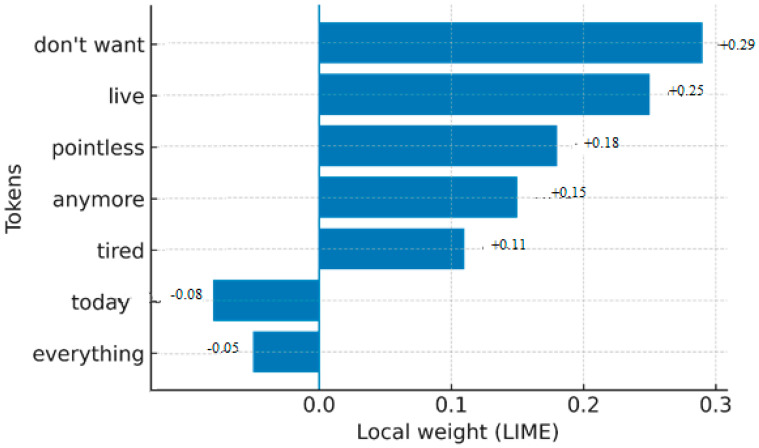
LIME-based explanation of a post, showing token contributions toward the suicidal label.

Furthermore, LIME was employed to provide complementary post-level interpretability using locally faithful surrogate models. As depicted in Figure 14 and summarized in Table 17, LIME identified tokens such as “don’t want,” “live,” and “pointless” as strong positive contributors toward a suicidal classification, whereas neutral terms such as “everything” exerted minimal influence. The combined use of SHAP and LIME supports transparency and interpretability of the proposed framework.

### 5.10. Training Dynamics and Convergence Analysis

**Figure 15:**
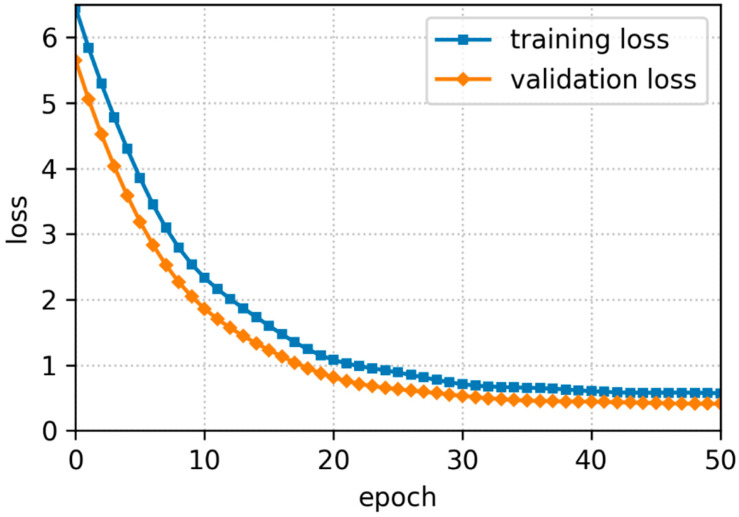
Training and validation loss curves of the GraphSAGE module over 50 epochs after BERT-CNN feature extraction.

As a further measure to confirm training stability and generalization behavior, we report the cross-entropy loss curves for the training and validation splits. The proposed model is trained in a two-step manner: first, the BERT-CNN module is fine-tuned 5 times for post-level classification; then, the output of the BERT-CNN module is used as node representations, and the GraphSAGE module is trained 50 times for graph-based post-level refinement and community-level reasoning. Figure 15 shows the training and validation loss of the GraphSAGE stage only. The training loss decreases monotonically, while the validation loss also declines and gradually plateaus, indicating stable convergence. The low difference between the training and validation loss suggests that there is no overfitting during the GraphSAGE training (using the chosen dropout regularization and user-disjoint testing protocol).

### 5.11. Dataset Statistics, Annotation Protocol, and Experimental Setup

We evaluated the proposed hybrid framework for risk detection on two benchmark datasets that are based on the Reddit social media platform and include posts with neutral, depressed and suicidal ideation, namely the eRisk benchmark dataset and the CLPsych Shared Task dataset. For clarity, the suicidal ideation labels are derived from CLPsych only, and the eRisk dataset provides only the Neutral and Depressed classes under the new label mapping. Table 18 shows that the data were split into 70% training, 15% validation and 15% testing.

Suicidal ideation labels are derived exclusively from CLPsych; the eRisk dataset does not include such annotations, and therefore no suicidal samples are counted from eRisk in this study.

#### 5.11.1. Annotation Protocol and Label Reliability

This work is based solely on the official labels of the benchmark datasets eRisk and CLPsych and does not involve any re-annotation or label modification. The eRisk dataset was curated within the CLEF eRisk shared-task framework and provides user-level labels for mental health conditions such as depression, based on longitudinal user timelines. The CLPsych data were released as part of shared tasks with expert-driven annotation methodologies detailed in original task descriptions. For the sake of avoiding unfounded specificity, we do not claim separate certification status, or ranges on inter-annotator agreement other than those cited in the original publications. Inter-annotator agreement varies across annual tasks and is reported in the original CLPsych publications rather than being generalized. These datasets provide curated and clinically informed labels, supporting reliable model training and evaluation under standardized benchmark conditions.

Experiments were conducted on a workstation with an NVIDIA RTX A6000 GPU 48 GB VRAM, Intel Xeon Gold 6326 CPU, 128 GB RAM, and Ubuntu 22.04 LTS. Models were implemented in PyTorch 2.0 using Hugging Face Transformers 4.30 and PyTorch Geometric 2.4. The main settings were: BERT bert-base-uncased, max sequence length 128, 5 epochs, learning rate 2 × 10^−5^; CNN with 1D convolution filters of sizes 3, 4, and 5, 100 filters each, and dropout 0.5; SBO with population size 30, 50 iterations, and fitness = accuracy − λ × selected features; and a two-layer GraphSAGE model with hidden dimension 128 and dropout 0.3.

#### 5.11.2. User-Disjoint Evaluation Protocol

A strict user-disjoint data splitting strategy was used to ensure realistic performance estimation and avoid information leakage in all experiments. The user posts were limited to a single split (training, validation, or testing), so that no user is represented in more than a single subset. The protocol is of particular significance to mental health detection tasks where linguistic patterns are very user-specific and post-level splitting can cause overestimation of performance. Any reported outcomes are in line with this user-level separation, which is the best practice for analyzing mental health based on social media.

## 6. Conclusions and Future Work

This paper introduced a hybrid text-based system to identify mental health risk signals in social media posts through bio-inspired feature selection, transformer-based language modeling, and graph-based relational analysis. The model incorporates Secretary Bird Optimization (SBO) to select salient linguistic and psychological features, a BERT-CNN model for post-level classification, and a Graph Neural Network (GraphSAGE) to learn user–post relationships.

Strict user-disjoint experiments using the eRisk and CLPsych benchmark datasets demonstrate that the proposed framework achieves higher accuracy, F1-score, and ROC-AUC compared to classical machine learning and strong deep learning baselines. The SBO module improves generalization by reducing feature redundancy, while the GNN module enhances post hoc community-level analysis. SHAP and LIME further support interpretability by highlighting linguistically and psychologically relevant indicators, with consistent behavior observed across datasets and calibration analysis.

This study focuses on post-level and community-level risk detection rather than temporal early risk prediction or real-time intervention. As such, the proposed system should not be taken as a clinical diagnostic system, but rather a research-focused decision support system for high-throughput screening. The “human-in-the-loop” approach is theoretical and supported by explainability techniques (SHAP/LIME), but not implemented in the current work.

Future work will extend this framework to longitudinal and temporal modeling, multilingual and cross-platform adaptation, and real-time screening pipelines. We will also implement “uncertainty-aware” decision thresholds and clinician-driven validation for safe use. Also, we plan to release code, pre-processing, and model settings to facilitate replication and extensions.

## Figures and Tables

**Table 1:** Summary of related studies on depression and suicide risk detection, including methods applied and their reported limitations.

S. No.	Year	Author(s)	Ref.	Algorithm/Strategy Used	Key Limitation/Scope
1	2022	Yeow & Chua	[16]	SVM with linguistic features + lexicon-based sentiment	Limited contextual understanding; struggles with figurative language
2	2013	De Choudhury et al.	[17]	Behavioral and interaction-based Reddit signals	Task-specific behavioral cues; limited deep semantic modeling
3	2024	Chopra et al.	[18]	CNN/LSTM hybrid architectures	Limited bidirectional contextual modeling compared to transformers
4	2024	Gupta	[19]	BERT-based contextual modeling	Primarily post-level modeling; limited relational reasoning
5	2023	Inamdar et al.	[20]	NLP-based stress detection	Limited modeling of post–user relationships
6	2024	Razavi et al.	[21]	ML/DL-based mental health analysis	Limited integration of optimization, graph reasoning, and reliability analysis
7	2019	Zakaria et al.	[22]	StressMon passive sensing system	Not focused on social media text; limited community-level modeling
8	2024	Zhai et al.	[23]	Domain-adaptive MentalBERT	Strong contextual modeling; limited graph/community reasoning
9	2025	Taiwo & Al-Bander	[24]	Transformer-based distress detection	Cross-dataset generalization requires further validation
10	2025	Hasan et al.	[25]	Transformer vs. LSTM comparison	Focused on model comparison; limited relational modeling
11	2025	Karami	[26]	Graph convolutional modeling for depression	Graph-based modeling present; limited integration with contextual encoders and feature optimization
12	2024	Liu et al.	[27]	Spatio-temporal graph neural networks	Focus on temporal dynamics; different data assumptions
13	2024	Al-Shalif et al.	[28]	Meta-heuristic feature selection	Limited integration with deep contextual models
14	2024	Fu et al.	[29]	Secretary Bird Optimization (SBO)	General-purpose optimizer; not tailored to mental health text pipelines
15	2023	Dwivedi et al.	[30]	SHAP/LIME explainability methods	Explainability only; not a full detection pipeline
16	2024	Dhurandhar et al.	[31]	Model-agnostic explanations	Focus on interpretability; limited domain-specific evaluation
17	2017	Lundberg & Lee	[32]	SHAP unified explanation framework	General-purpose method; requires task-specific adaptation
18	2025	Putica et al.	[33]	Ethical AI decision-making	Conceptual framework; limited technical implementation
19	2024	Mosa et al.	[34]	Privacy and surveillance ethics	Limited operational deployment details
20	2025	Harrington	[35]	Fairness and bias in NLP	General fairness analysis; limited mental health application
21	2024	Sadeghi et al.	[36]	Multimodal depression detection (speech + facial cues)	Requires non-text modalities; limited applicability to text-only datasets
22	2025	Wang et al.	[37]	Cross-modal co-occurrence analysis	Modality constraints; limited use in text-only scenarios
23	2024	Yan et al.	[38]	Longitudinal suicide risk modeling	Temporal focus; limited graph-based community reasoning
24	2025	Jacob	[39]	Bi-LSTM depression detection	Sequential modeling; limited contextual and relational modeling
25	2024	Sao & Lim	[40]	Transfer learning (MIRoBERTa)	Domain bias may persist; limited community reasoning
26	2024	Lin et al.	[41]	Adversarial domain adaptation	Requires diverse data; higher deployment complexity
27	2024	Al-Remawi et al.	[42]	ML-based suicide prediction review	Evaluation varies across datasets
28	2025	Gkintoni et al.	[43]	Digital mental health systems	Focus on intervention; not detection modeling

**Table 2:** Label harmonization from eRisk and CLPsych to the unified 3-class label space.

Dataset	Original Label Name (as Released)	Unified Class Used in This Paper
eRisk	Control/Non-risk (exact dataset label)	Neutral
eRisk	Depression-positive (exact dataset label)	Depressed
eRisk	No explicit suicide-related label in original dataset	Not applicable (no mapping)
CLPsych	No-risk/Control	Neutral
CLPsych	Depression-related (if present)	Depressed
CLPsych	Suicide risk/Self-harm (or risk levels collapsed)	Suicidal Ideation

Note that the original eRisk dataset does not include suicidal labels and therefore no labels are inferred or created. All suicide-related labels used in this study are from the CLPsych dataset.

**Table 3:** User-level statistics required for user-disjoint validation.

Dataset	Total Users	Train Users (70%)	Val Users (15%)	Test Users (15%)	Median Posts/User (IQR)
eRisk (CLEF 2017/2018)	887	621	133	133	10 (8–12)
CLPsych (Shared Task 2015–2019)	1241	869	186	186	6 (4–8)

**Table 4:** Examples of annotated social media posts from eRisk and CLPsych datasets used for training.

Post ID	Dataset	Platform	Post Excerpt	Label
P001	eRisk	Reddit	“I haven’t been able to get out of bed for days…”	Depression
P045	CLPsych	Reddit	“I feel like I’m drowning every time I go to sleep.”	Depression
P102	CLPsych	Reddit	“Today I seriously thought about ending it all.”	Suicidal Ideation
P130	CLPsych	Reddit	“Just needed to vent… not doing great mentally.”	Depression
P210	eRisk	Reddit	“I’m just tired of pretending like everything is okay.”	Depression
P305	CLPsych	Reddit	“Sometimes I just want to disappear forever.”	Suicidal Ideation
P400	eRisk	Reddit	“Had a great time with friends today!”	Neutral

**Table 5:** Handcrafted feature pool and SBO configuration.

Component/Setting	Notation	Value Used	Notes (for Reproducibility)
TF-IDF word n-grams	$N_{\mathrm{tfidf}\;}$	5000	n-gram range (1,2), max_features =5000; fit on train only
LIWC categories	$N_{\mathrm{liwc}\;}$	93	standard LIWC category proportions
POS/style ratios	$N_{\mathrm{pos}\;}$	12	e.g., pronoun/verb/noun/adj/adv ratios, negation, punctuation ratios
Sentiment features	$N_{\mathrm{sent}\;}$	5	e.g., polarity/compound and/or pos/neg/neu + subjectivity
Engineered feature total	V_eng_	110	V_eng_ = V_iwc_ + V_pos_ + V_sent_
Total handcrafted pool size	N	5110	N=5000+93+12+5
Inner CV folds (train-only)	K	5	used in ${\mathrm{Acc}}_{\mathrm{inner}\text{-}\mathrm{Cv}\;}$
Fitness penalty weight	λ	0.01	light penalty to prefer compact subsets
SBO population size	P	30	number of candidate masks
Max iterations	T	50	stopping criterion (or convergence)
Selected features (SBO)	(	S* = 45	)
Selected features (RF baseline)		73	used for reduction comparison
Selected-feature reduction	Equation (3)	38.36%	(73−45)/73

**Table 6:** Top 20 features selected using SBO from the initial feature pool (TF-IDF, sentiment, LIWC).

Rank	Feature Name	Type	Description
1	tfidf_suicide	TF-IDF	Term frequency of the word “suicide”
2	tfidf_die	TF-IDF	Frequency of the word “die”
3	tfidf_tired	TF-IDF	Term frequency of emotional fatigue indicator
4	liwc_sadness	LIWC	Sadness-related lexical count
5	sentiment_neg	Sentiment Score	Negative polarity (VADER/TextBlob)
6	liwc_self_focus	LIWC	Use of 1st person singular pronouns
7	tfidf_lonely	TF-IDF	Occurrence of the word “lonely”
8	sentiment_subjectivity	Sentiment Score	Degree of subjectivity
9	tfidf_hurt	TF-IDF	Emotional expression of pain
10	liwc_anger	LIWC	Anger category words (e.g., hate, rage)
11	tfidf_empty	TF-IDF	Expressing emotional void
12	pos_pronoun_ratio	POS Ratio	Pronoun usage frequency
13	tfidf_worthless	TF-IDF	Despair/self-value marker
14	tfidf_sleep	TF-IDF	Reference to sleep disturbance
15	liwc_anxiety	LIWC	Anxiety-related lexical score
16	tfidf_cut	TF-IDF	Self-harm indication term
17	tfidf_sad	TF-IDF	Basic emotional term frequency
18	tfidf_help	TF-IDF	Request or reference to needing help
19	sentiment_compound	Sentiment Score	Compound emotional sentiment score
20	tfidf_lost	TF-IDF	Expression of confusion or helplessness

**Table 7:** Comparison of model performance with baseline approaches.

Model	Accuracy (%)	Precision	Recall	F1-Score	ROC-AUC
Logistic Regression	78.4	0.69	0.63	0.66	0.741
SVM (linear kernel)	80.2	0.72	0.64	0.68	0.759
BERT (fine-tuned)	88.5	0.83	0.85	0.84	0.902
BERT + CNN	90.3	0.86	0.88	0.87	0.918
SBO + BERT + CNN	91.2	0.88	0.89	0.88	0.926
Full Pipeline (Ours)	93.1	0.91	0.92	0.91	0.944

**Table 8:** User-Level Risk Detection Performance.

Aggregation Method	Accuracy (%)	F1-Score	ROC-AUC
Mean Probability	91.8	0.89	0.936
Maximum Probability	90.6	0.87	0.921
Majority Vote	89.4	0.86	0.914

**Table 9:** Per-dataset post-level test performance of the proposed full pipeline.

Dataset (Test Set)	Accuracy (%)	Precision	Recall	F1-Score	ROC-AUC
eRisk	92.8	0.90	0.91	0.90	0.941
CLPsych	93.4	0.91	0.92	0.91	0.947

**Table 10:** Class-wise post-level performance of the proposed full pipeline.

Class	Precision	Recall	F1-Score
Neutral	0.93	0.95	0.94
Depression	0.90	0.91	0.90
Suicidal Ideation	0.89	0.90	0.89

**Table 11:** Test set reliability over five seeds (mean ± SD) for Macro-F1 and suicidal recall.

Metric (Test Set)	Mean ± Std (5 Seeds)
Macro-F1	0.910 ± 0.006
Suicidal Recall	0.900 ± 0.011

**Table 12:** Operating point analysis for suicidal risk screening at different probability thresholds.

Threshold (t)	Suicidal Recall	Suicidal Precision	False Alarms/1000 Posts	NNR = 1/Precision
0.30	0.95	0.62	38	1.61
0.50	0.90	0.74	22	1.35
0.70	0.82	0.84	11	1.19

**Table 13:** Ablation study showing performance variations across component combinations.

Model Variant	Accuracy (%)	F1-Score	ROC-AUC
BERT Only	88.5	0.84	0.902
BERT + CNN	90.3	0.87	0.918
BERT + CNN + GNN	91.4	0.89	0.929
SBO + BERT + CNN	91.2	0.88	0.926
Proposed Method	93.1	0.91	0.944

**Table 14:** Comparison of selected feature count and classification performance for SBO vs. traditional selectors.

Feature Selection Method	Features Selected	Accuracy (%)	F1-Score	ROC-AUC
Random Forest Importance	73	89.5	0.86	0.913
Secretary Bird Optimization (SBO)	45	91.2	0.88	0.926

**Table 15:** Community statistics and node-level classification accuracy using GNN.

Metric	Value
Node Classification Accuracy	91.4%
Average Community Size	27.6 nodes
Number of High-Risk Communities	14
Average Internal Edge Density	0.61
Modularity (Louvain Method)	0.73

**Table 16:** Top 10 SHAP-ranked features across suicidal ideation posts.

Rank	Feature	Type	Mean SHAP Value
1	“die”	Lexical (TF-IDF)	0.142
2	Sadness (LIWC)	Affective Feature	0.134
3	Negative Polarity Score	Sentiment	0.127
4	“end it”	Lexical Phrase	0.121
5	Self-focus (LIWC)	Affective Feature	0.114
6	“goodbye”	TF-IDF	0.107
7	“worthless”	TF-IDF	0.102
8	Anxiety (LIWC)	Affective Feature	0.096
9	Verb Tense (past)	Linguistic Style	0.091
10	“can’t breathe”	Crisis Phrase	0.085

**Table 17:** LIME-identified token contributions for a suicidal ideation post.

Token	Contribution (Weight)	Effect on Prediction
“don’t want”	+0.29	Pushes toward suicidal
“live”	+0.25	Pushes toward suicidal
“pointless”	+0.18	Pushes toward suicidal
“anymore”	+0.15	Pushes toward suicidal
“everything”	–0.05	Neutral contribution

**Table 18:** Dataset statistics and user-disjoint train/validation/test splits for the eRisk benchmark dataset and CLPsych Shared Task dataset.

Class Label	eRisk	CLPsych	Total	Train	Val	Test
Neutral	5200	4300	9500	6650	1425	1425
Depressed	3100	2900	6000	4200	900	900
Suicidal Ideation	0	1700	1700	1190	255	255
Total	8300	8900	17,200	12,040	2580	2580

## Data Availability

The datasets used in this study are publicly available Reddit-based benchmark datasets, namely eRisk and the CLPsych Shared Task dataset. The Kaggle links provide accessible mirrored versions of these real-world benchmark datasets: eRisk, https://www.kaggle.com/datasets/albertouah/erisk-feature-engine-v2 (accessed on 15 November 2025)**;** and CLPsych, https://www.kaggle.com/datasets/sagarikashreevastava/cognitive-distortion-detetction-dataset (accessed on 10 January 2026). Reproducibility scripts and preprocessing settings are available from the corresponding author upon reasonable request.
